# Cytokine release and NLRP3 inflammasome activation induced by low-abundance oral bacterial biofilms

**DOI:** 10.1080/20002297.2025.2552167

**Published:** 2025-08-27

**Authors:** Maribasappa Karched, Radhika Guleri Bhardwaj, Manal Abu Al-Melh, Muawia Abdalla Qudeimat

**Affiliations:** a Department of Bioclinical Sciences, College of Dentistry, Kuwait University, Jabriya, Kuwait; b Department of Biotechnology, School of Arts & Science, American International University, Jahra, Kuwait; c Department of Developmental and Preventive Sciences, College of Dentistry, Kuwait University, Jabriya, Kuwait

**Keywords:** LAB species, oral, Inflammation caspase-1, NLRP3, cytokines, biofilms, *Neisseria flavescens*, *Cardiobacterium hominis*

## Abstract

**Background:**

Low-abundance bacterial (LAB) species, despite their low prevalence, may contribute to oral inflammatory diseases by triggering host immune responses. The NLRP3 inflammasome plays a key role in inflammation, but its activation by LAB species remains unclear.

**Aim:**

This study examined whether selected LAB species and their biofilm-secreted components induce cytokine production and inflammasome activation in human peripheral blood mononuclear cells (PBMCs).

**Methods:**

Biofilms of selected LAB species were established, and supernatants were collected. PBMCs were stimulated with biofilms or supernatants, and cytokine levels were quantified using ELISA. The expression of NLRP3 and Caspase-1 genes was analyzed through real-time PCR.

**Results:**

Biofilms induced significantly higher levels of pro-inflammatory cytokines (TNF-α, IL-6, IL-1β, and IL-18) compared to supernatants, with C. hominis, N. flavescens, and D. pneumosintes being the most potent inducers. Biofilms also led to a marked increase in NLRP3 expression, while supernatants primarily activated Caspase-1 expression, indicating distinct inflammasome activation pathways.

**Conclusions:**

These findings highlight the immunostimulatory potential of LAB species, particularly their ability to activate NLRP3 and drive inflammation. The differential activation of NLRP3/Caspase-1 by biofilms and supernatants suggests distinct pathogenic mechanisms. Targeting such mechanisms/pathways could offer new therapeutic strategies to mitigate inflammation linked to oral infections.

## Introduction

The oral cavity harbors a complex and dynamic microbial ecosystem, where bacterial interactions are shaped by distinct ecological conditions [1,2]. While significant progress has been made in understanding host–microbe interactions, research has predominantly focused on high-abundance species due to technological limitations that historically hindered the detection and characterization of low-abundance taxa (typically comprising < 0.1–1% of relative abundance) [3–5]. Early microbiome studies, relying on 16S rRNA gene sequencing, often filtered out these low-abundance taxa during quality control steps, as their rarity made them difficult to distinguish from sequencing noise or contamination [6]. However, advancements in sequencing technologies and bioinformatics have revealed that low-abundance bacteria exhibit remarkable taxonomic diversity and functional potential, underscoring their potential importance in host–microbe interactions [7–10].

Recent research has shifted toward investigating low-abundance microorganisms, demonstrating that their rarity does not diminish their significance. The concept of ‘keystone species’ suggests that even minor community members can exert a disproportionate influence on microbial ecology and host health [6,11]. For example, Eriksson et al. (2017) used PacBio SMRT amplicon sequencing to study tooth biofilm microbiota and identified a statistically significant difference: *Dialister pneumosintes* was absent in caries-free individuals but present at 0.03% abundance in caries-active ones [7]. These findings highlight the importance of examining low-abundance bacteria alongside dominant taxa to understand host–microbe interactions fully.

In the oral cavity, the core microbiome consists of species adapted to this niche, benefiting from favorable nutrient availability and oxygen levels. However, low-abundance opportunistic pathogens may disrupt microbial homeostasis and drive dysbiosis. For example, *Porphyromonas gingivalis*, a well-characterized periodontitis-associated pathogen, can alter microbial community structure and trigger host inflammation despite its low-abundance [12]. Similar trends have been observed beyond the oral cavity, where low-abundance bacteria are implicated in inflammatory diseases such as inflammatory bowel disease, rheumatoid arthritis, and metabolic disorders [13–16]. Conversely, some low-abundance species, such as *Bifidobacterium*, contribute to microbial homeostasis by modulating immune responses and preventing pathogen colonization [17]. These findings underscore the dual role of low-abundance bacteria in health and disease.

The pyrin domain-containing-3 (NLRP3) inflammasome is a key mediator of inflammation, activated in response to diverse stimuli, including pathogen-associated molecular patterns (PAMPs) [18,19]. The NLRP3 inflammasome is a multiprotein complex comprising the NLRP3 scaffold, the adaptor protein ASC (apoptosis-associated speck-like protein containing a caspase recruitment domain), and caspase-1. Upon activation, it facilitates the cleavage and secretion of pro-inflammatory cytokines, such as interleukin-1β (IL-1β) and IL-18 [20] through caspase-1-mediated proteolytic processing. While its activation has been extensively studied in response to high-abundance pathogens, its role in low-abundance bacterial interactions remains largely unexplored. In the oral cavity, evidence suggests that specific bacterial species can activate NLRP3, contributing to inflammatory cytokine production and immune responses [21]. Given the potential of low-abundance bacteria to function as keystone species, elucidating their role in NLRP3-mediated inflammation is crucial for understanding the mechanisms underlying oral inflammatory diseases [21]. Therefore, the aim of this study was to investigate whether select low-abundance bacterial species and their secreted components can induce cytokine production from human peripheral blood mononuclear cells (PBMCs). In addition, we explored the involvement of NLRP3 and caspase-1 in cytokine production triggered by low-abundance bacteria, providing insights into their potential contribution to oral inflammatory diseases.

## Materials and methods

The study was conducted according to the guidelines of the Declaration of Helsinki and approved by the Health Science Center Ethical Committee, Kuwait University (VDR/EC/3413). Informed consent was obtained from the volunteer who donated blood for the study.

### Bacteria and culture conditions

The selection of low-abundance bacterial strains for this study was based on operational taxonomic units (OTUs) identified in a previous study by Eriksson et al. (2017) [7]. All study species in the study were below a relative abundance cut-off of <1%. In that study, investigators analyzed bacterial communities from saliva and tooth biofilm using 16S rDNA sequencing with Illumina MiSeq (V3–V4 region) and PacBio SMRT (V1–V8 region), finding no significant differences in microbiota composition between the two sample types [7]. The list of bacterial strains used in this study is presented in Table 1. All strains were revived from stocks frozen at −80°C. The strains were cultured on brucella blood agar in anaerobic atmosphere or in 5% CO_2_ in air at 37°C for 3–5 days.

**Table 1.** LAB bacterial species used in this study.

1. *Actinomyces viscosus* CCUG 14476
2. *Actinomyces odontolyticus* CCUG 20536
3. *Cardiobacterium hominis* CCUG 2711
4. *Gemella morbillorum* CCUG 18164
5. *Neisseria flavescens* CCUG 345
6. *Dialister pneumosintes* CCUG 21025
7. *Parvimonas micra* ATCC 33270
8. *Eikennela corrodens* CCUG 2138
9. *Dialister invisus* CCUG 47026
10. *Filifactor alocis* CCUG 47790

### Biofilm cultures

Biofilm cultures were standardized by inoculating OD_600_-adjusted suspensions (OD_600_ = 1.0) to ensure consistent initial microbial loads across experiments [22]. The total number of cells per ml at OD_600_ = 1 as enumerated by using Neubauer counting chamber (Celeromics, Cambridge, UK) were for *Actinomyces viscosus* 2.5 × 10^8^, *Actinomyces odontolyticus* 4.2 × 10^8^, *Cardiobacterium hominis* 4.1 × 10^8^, *Gemella morbillorum* 6 × 10^8^, *Neisseria flavescens* 1.09 × 10^9^, *Dialister pneumosintes* 1.02 × 10^9^, *Parvimonas micra* 1.1 × 10^9^, *Eikenella corrodens* 8.4 × 10^8^, *Dialister invisus* 1 × 10^9^ and *Filifactor alocis* 5.2 × 10^8^.

Briefly, bacterial strains were grown on Columbia Blood Agar (CBA) for 2 days before the biofilm setup. Colonies were harvested using a sterile cotton swab, suspended in Brucella broth, and adjusted to an optical density of OD_600_ = 1 in the same broth. A 100-μl aliquot of each strain from an OD_600_ = 1 suspension was inoculated into respective wells containing 900 μl brucella broth supplemented with 0.001% pyridoxal. The plates were incubated at 37°C in 5% CO_2_ in air or in anaerobic atmosphere for 2 days to allow biofilm formation. Wells containing broth without bacteria served as negative controls.

### Biofilm quantification assay

After 24 hours of biofilm growth, the broth supernatant was removed, and the biofilms were gently washed with 1 mL of sterile PBS. For visual assessment, the biofilms were stained with 1 ml of 2% crystal violet for 10 minutes, rinsed six times with PBS, air-dried, and photographed. For absorbance-based quantitative analysis, biofilms were fixed with methanol (1 ml/well, 15 min), air-dried (45 min), and stained with 0.1% crystal violet (1 ml/well, 20 min). Excess stain was removed by washing five times with tap water, followed by destaining with 500 μl of 33% acetic acid (5 min shaking). The absorbance of the solubilized dye was measured at 590 nm using an iMark™ Microplate Absorbance Reader (Bio-Rad, Hercules, CA, USA).

### Isolation of human peripheral blood mononuclear cells (PBMCs)

PBMCs were isolated from the blood of a systemically healthy human volunteer. Blood was collected by venipuncture into heparin-containing Vacutainer tubes (4 ml per tube). PBMCs were then isolated using the Ficoll-Paque density gradient centrifugation method as described earlier [23]. Under sterile conditions, PBMCs were isolated by a density gradient centrifugation (3400 rpm, 10–12 min). The buffy coat layer containing PBMCs was collected, washed twice with RPMI medium, and centrifuged (2000 rpm, 5 min) to pellet the cells. The pellet was then resuspended in 1 ml of RPMI medium supplemented with 10% heat-inactivated fetal bovine serum and 2% antibiotic-antimycotic solution. The cell concentration was determined on a hemocytometer.

### Stimulation of PBMCs with biofilms

After the cell concentrations were determined, the PBMCs briefly centrifuged as above and resuspended in the RPMI medium without antibiotics were stimulated with live static biofilms for 24 hours. To achieve this, the broth supernatants from 2-day-old biofilm cultures were removed, and the static biofilms in the wells were washed once with sterile phosphate-buffered saline (PBS). PBMCs (0.5 ml, 10^6^ cells/ml) were then added to the biofilms in each well and incubated for 24 hours at 37°C in 5% CO_2_ in air for stimulation. A well containing only PBMCs, without biofilm, served as the negative control.

### Stimulation of PBMCs with biofilm-supernatants

PBMCs were stimulated for 24 hours with extracellular components from biofilm supernatants. To prepare the supernatants, broth from 2-day-old biofilms was homogenized by vigorous vortexing for 30 seconds, filtered through 0.2 μm syringe filters to remove bacterial cells, and aliquoted (100 μL per well). This volume was optimized to maintain PBMC viability while ensuring detectable cytokine responses within ELISA linear ranges. Subsequently, 0.5 mL of PBMCs (10^6^ cells/mL) was added to each well. The plates were incubated at 37°C in 5% CO_2_ in air for 24 hours. Broth without bacteria served as a negative control.

### ELISA quantification of selected cytokines

The following cytokines were selected to test the immune responses and inflammation induced by the study bacteria: TNF-α, IL-6, IL-10, IL-1β, and IL-18. The cytokines were quantified using Quantikine® ELISA Immunoassay Kits as per manufacturer’s instructions (R&D Systems, Minneapolis, MN, USA). Standards and samples were added to the wells, allowing the target cytokine to bind to the immobilized antibody. After washing away unbound substances, an enzyme-linked polyclonal antibody specific to the cytokine was introduced. Subsequent washes removed any unbound antibody-enzyme complex, followed by the addition of a substrate solution that produced a color change. The reaction was stopped, and the color intensity – directly proportional to the cytokine concentration – was measured using an iMark™ Microplate Reader (Bio-Rad, Hercules, CA, USA).

### ELISA quantification of NLRP-3 and Caspase-1

NLRP-3 and Caspase-1 were quantified using the kits from Bio-Techne (Minnesota, USA) according to manufacturer’s instructions. After washing cells with cold PBS, the pellets were lysed and centrifuged, and the supernatants were stored at −20°C until used for assays. For the ELISA, samples and standards were incubated in wells (2 h, 37°C), followed by sequential additions of biotin-conjugated antibody (1 h, 37°C), streptavidin-HRP (30 min, 37°C), TMB substrate (15 min, dark) and stop solution. In the case of Caspase-1, after incubating with samples and standards, the wells were treated with Caspase-1 antiserum, Caspase-1 conjugate, substrate, and stop solutions and were added sequentially with intermittent washings. The absorbance was immediately measured at 450 nm using the iMark^TM^ Microplate Reader (Bio-Rad).

### RNA extraction from stimulated PBMCs

Total cellular RNA was extracted from these stimulated PBMCs using the QIAamp RNA Blood Mini Kit (QIAGEN, Germantown, MD, USA), following the manufacturer’s instructions. After 24 hours of incubation of PBMCs with biofilms and biofilm-supernatants, cells were treated with lysis buffer, and precipitated using 70% ethanol. The samples were then transferred to a QIAamp spin column, centrifuged, followed by sequential washing of bound RNA. The RNA was then eluted with 40 μl of RNase-free water at 8000 × g for 1 minute. The concentration of the eluted RNA was measured using a NanoDrop^TM^ 1000 spectrophotometer, and its purity was evaluated by the A260/A280 ratio.

### Synthesis of cDNA

The purified RNA was reverse-transcribed into cDNA using the High-Capacity cDNA Reverse Transcription Kit (Applied Biosystems, Leicestershire, UK) following the manufacturer’s instructions. A 20-µl reaction mixture was prepared by combining 10 µl of RNA with an RT master mix containing dNTPs, random primers, MultiScribe^TM^ reverse transcriptase, and nuclease-free water. The reaction was incubated in a thermal cycler under the following conditions: 25°C for 10 min, 37°C for 120 min, 85°C for 5 min, and a final hold at 4°C. The resulting cDNA was then quantified using a NanoDrop 1000, and its purity was assessed via the A260/A280 ratio.

### Real-time PCR to determine relative mRNA expression of NLRP3 and Caspase-1 genes

The primers used in this study are listed in Table 2. A reaction mixture of 20 μl, comprising 4 μl of 5× HOT FIREPol EvaGreen qPCR Supermix (Solis Biodyne), 0.5 μl each of forward and reverse primers (0.25 µM), 14 μl nuclease-free water, and 1 μl cDNA template (~20 ng) was used for real-time RT-PCR on the ABI 7500 FAST Real-Time PCR System. The thermal profile used was as follows: initial denaturation at 95°C for 10 minutes, followed by 40 cycles of denaturation at 95°C for 15 seconds, annealing at 55°C for 30 seconds, and extension at 72°C for 30 seconds, with data acquisition at the extension step. Primer sequences for *nlrp3* and *caspase-1* are provided in Table 2. The GAPDH gene (encoding Glyceraldehyde-3-phosphate dehydrogenase) was used as the housekeeping gene.

**Table 2.** Primers used in this study.

Primer Sequence	Reference
NLRP3-F: 5′-AGCCCCGTGAGTCCCATTA-3′ NLRP3-R: 5′-ACGCCCAGTCCAACATCATCT-3′	Su et al. 2022 [48]
Caspase-1-F: 5′-TGAATACCAAGAACTGCCCAAG-3′ Caspase-1-R: 5′-GCATCATCCTCAAACTCTTCTGTAG-3′	Su et al. 2022 [48]
GAPDH-F: 5′-CCA CTC CTC CAC CTT TGA C-3′ GAPDH-R: 5′-ACC CTG TTG CTG TAG CCA-3′	Xue et al. 2015 [49]

### Data normalization

**Figure 1.**
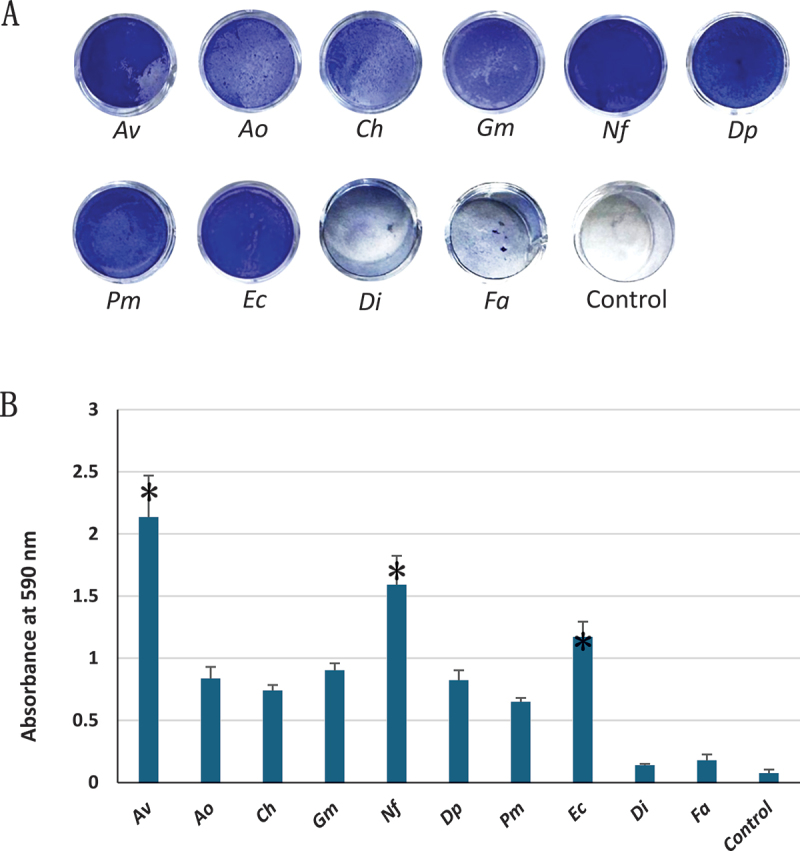
Crystal violet staining and quantification of LAB biofilms. Biofilms were grown in brucella broth for 24 h, washed with PBS, and stained with 2% crystal violet for imaging or 0.1% crystal violet for quantification. For quantification, fixed biofilms were solubilized in 33% acetic acid, and absorbance was measured at 590 nm.

To account for differences in biofilm biomass quantities among species, cytokine quantities and gene expression data were normalized against the biofilm quantities (Figure 1). This approach allowed consistent and reproducible comparisons across species, eliminating the possibility that observed differences in cytokine production were merely due to disparities in biofilm biomass. Cytokine quantities or gene expression values were divided by the corresponding crystal violet absorbance values of each biofilm. This yielded normalized cytokine concentration or gene expression data per unit of biofilm absorbance.

Av: *A. viscosus*, Ao: *A. odontolyticus*, Ch: *C. hominis*, Gm: *G. morbillorum*, Nf: *N. flaviscens*, Dp: *D. pneuosintes*, Pm: *P. micra*, Ec: *E. corrodens*, Di: *D. invisus*, Fa: *F. alocis*, CB: control broth.

### Statistics

One-way ANOVA was used to assess variations in cytokine and biomarker levels across different species. For gene expression analysis, normalized relative quantities were log-transformed, and differences in fold changes of gene expression across groups were evaluated using one-way ANOVA. Tukey’s post-hoc test was applied in all one-way ANOVA analyses to conduct multiple group comparisons. A p-value less than 0.05 was considered statistically significant. All the experiments had two biological replicates with two technical replicates each. Two independent experiments were performed. Statistical analyses were performed using the SPSS version 28 (IBM Corp., Chicago, IL, USA) on a Windows platform.

## Results

### LAB biofilm quantification

Visual inspection of the LAB biofilms after crystal violet staining revealed that *A. viscosus*, *N. flavescens*, *D. pneumosintes* and *E. corrodens* formed strong biofilms (Figure 1(A)). From absorbance measurements of the crystal violet staining (Figure 1(B)), *A. viscosus*, *N. flavescens*, and *E. corrodens* formed significantly higher biofilms compared to all other species (*p* < 0.001). On the other hand, *D. invisus* and *F. alocis* exhibited poor biofilm-forming ability which was significantly lower than the rest of the LAB species (*p* < 0.001).

### Cytokine production in PBMCs induced by biofilms and supernatants from LAB

**Figure 2.**
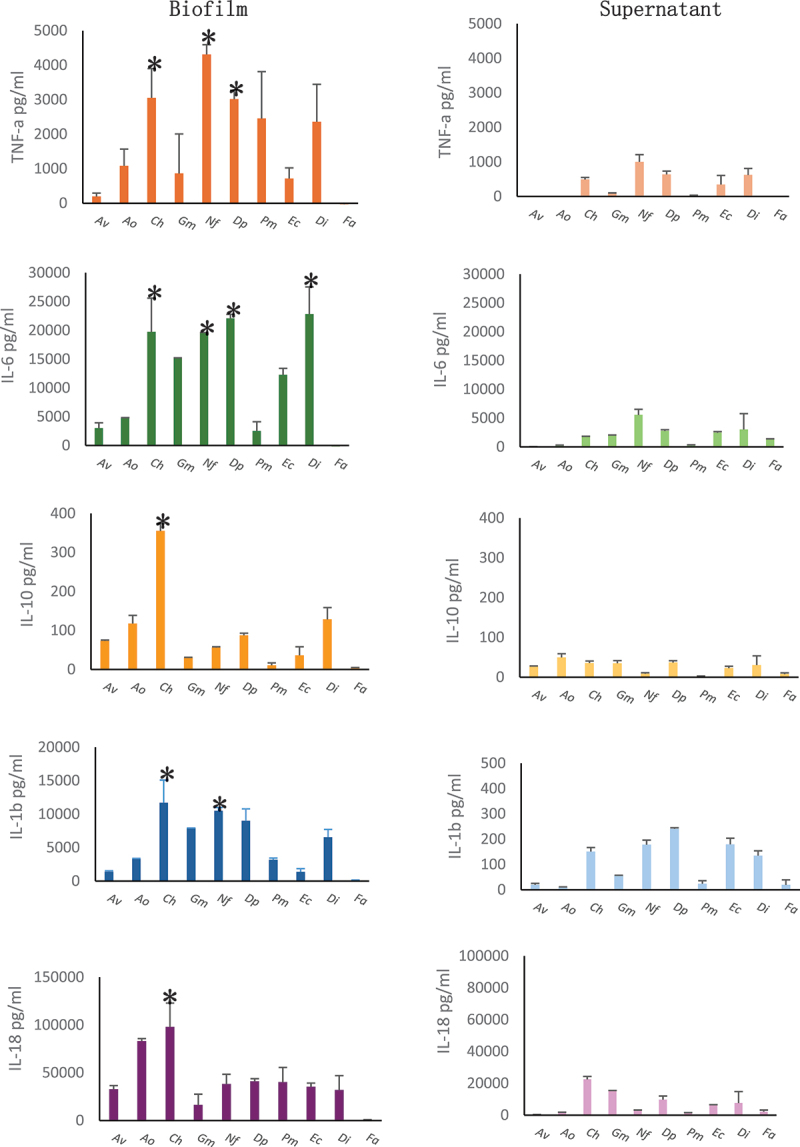
Stimulation of cytokine production from human PBMCs by LAB species. Biofilms and supernatants were used to induce cytokines from PBMCs. Following a 24-h incubation of the PBMCs with biofilms or supernatants, the stimulated samples were separated and subjected to cytokine quantification by using ELISA kits. Biofilms induced significantly higher cytokine levels than supernatants. *p < 0.001.

The data demonstrate that biofilms induce significantly higher levels of cytokines compared to supernatants, with *N. flavescens* and *C. hominis* being the most potent inducers in both cases, biofilm and supernatant (Figure 2). Mean ± SD (pg/ml) TNF-α levels were significantly (*p* < 0.001) higher in the biofilm, with *N. flavescens* (4312 ± 282), *C. hominis* (3053 ± 848 pg/ml), and *D. pneumosintes* (3015 ± 262) showing the highest production. In the supernatant, levels were much lower, with *N. flavescens* (1001 ± 209) still inducing highest amounts, but at reduced levels. *A. viscosus*, *A. odontolyticus*, and *F. alocis* showed no detectable TNF-α in the supernatant. For IL-6, biofilm levels were highest for *D. invisus* (22830 ± 4710), *D. pneumosintes* (22075 ± 704), *C. hominis* (19735 ± 5866), and *N. flavescens* (19625 ± 6) and while in the supernatant, levels were lower but still significantly higher (*p* < 0.05) for *N. flavescens* (5554 ± 982), *D. invisus* (3033 ± 2731), and *D. pneumosintes* (2767 ± 211). IL-10 levels in biofilms were moderate, with *C. hominis* (355 ± 18), *D. invisus* (128 ± 29), and *A. odontolyticus* (1217 ± 21) showing the highest production. In the supernatant, levels were lower, with *A. viscosus* (49 ± 9 pg/ml), *D. pneumosintes* (37 ± 4 pg/ml), and *C. hominis* (35 ± 5) being the highest inducers.

Av: *A. viscosus*, Ao: *A. odontolyticus*, Ch: *C. hominis*, Gm: *G. morbillorum*, Nf: *N. flaviscens*, Dp: *D. pneuosintes*, Pm: *P. micra*, Ec: *E. corrodens*, Di: *D. invisus*, Fa: *F. alocis*.

IL-1β levels were very high in biofilms for *C. hominis* (11700 ± 3393 pg/ml), *N. flavescens* (10511 ± 475 pg/ml) and, but lower in supernatants, with *D. pneumosintes* (241 ± 4 pg/ml) and *E. corrodens* (179 ± 24) still showing significant production (*p* < 0.05) (Figure 1). Finally, IL-18 levels in biofilms were high for *C. hominis* (98013 ± 24988 pg/ml), *A. odontolyticus* (83229 ± 2548 pg/ml), and *D. pneumosintes* (41022 ± 2778) while in supernatants, levels were lower but still notable for *C. hominis* (22481 ± 1865 pg/ml) and *G. morbillorum* (15242 ± 240 pg/ml).

### NLRP3 and Caspase-1 production from PBMCs

**Figure 3.**
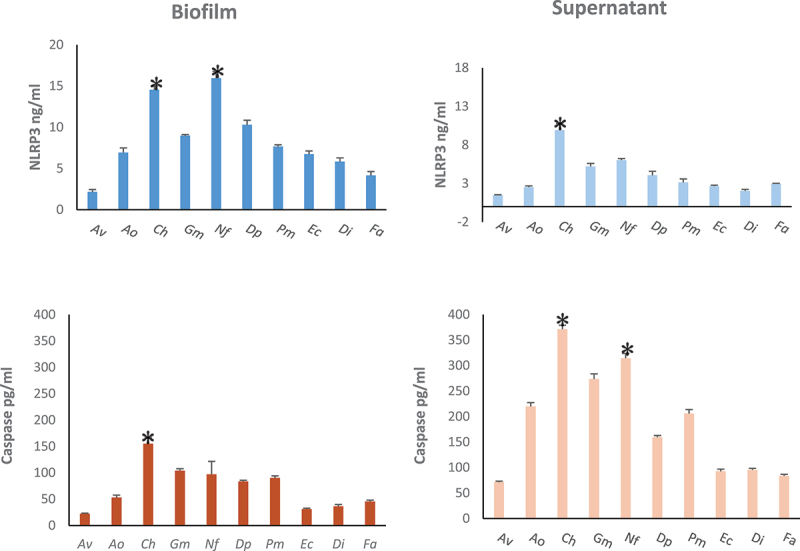
NLRP3 and Caspase-1 production from human PBMC. Biofilms and supernatants from LAB species were incubated with PBMCs for 24-h. NLRP3 and Caspase-1 were quantified using kits. NLRP3 levels were higher in biofilms than supernatants, with *N. flavescens* and *C. hominis* showing the greatest stimulation (*p* < 0.001). Caspase-1 levels were higher in supernatants than biofilms, with *N. flavescens* and *C. hominis* exhibiting the highest levels. Data are presented as mean ± standard deviation. *p < 0.001.

Significant differences were observed in NLRP3 and Caspase-1 levels between biofilm and supernatant conditions across the low-abundance bacterial species (Figure 3). For NLRP3, biofilms consistently induced higher levels compared to supernatants. In the biofilms, *N. flavescens* (15.9 ± 0.8 ng/ml) and *C. hominis* (14.5 ± 0.4 ng/ml) showed the highest NLRP3 levels (*p* < 0.001), indicating strong inflammasome activation. In contrast, the supernatants had lower NLRP3 levels, with *C. hominis* (9.9 ± 0.8 ng/ml) and *N. flavescens* (6 ± 0.32 ng/ml) and still being the highest, but significantly reduced compared to biofilms. For the other species, NLRP3 levels induced by biofilms were higher than those from supernatants, though the differences were less pronounced.

Av: *A. viscosus*, Ao: *A. odontolyticus*, Ch: *C. hominis*, Gm: *G. morbillorum*, Nf: *N. flaviscens*, Dp: *D. pneuosintes*, Pm: *P. micra*, Ec: *E. corrodens*, Di: *D. invisus*, Fa: *F. alocis*.

For Caspase-1, supernatants generally activated higher levels than biofilms, suggesting that secreted bacterial components are more effective at activating Caspase-1 expression. In supernatants, *C. hominis* (371 ± 7 ng/ml) and *N. flavescens* (314 ± 9.5 ng/ml) exhibited the highest Caspase-1 levels. In contrast, the biofilms of *C. hominis* (155 ± 11 ng/ml), *G. morbillorum* (104 ± 3.5) and *N*. *flavescens* (97 ± 24 ng/ml) also showed elevated levels but were significantly lower than in supernatants. A similar trend was observed for *A. viscosus* (biofilm: 22 ± 1.2 ng/ml; supernatant: 71 ± 1.8 ng/ml) and *A. odontolyticus* (biofilm: 53 ± 4.7 ng/ml; supernatant: 219 ± 7.5 ng/ml), where supernatants induced higher Caspase-1.

When comparing NLRP3 and Caspase-1 levels, biofilms were more potent activators of NLRP3 expression, while supernatants were more effective at activating Caspase-1.

### Expression of NLRP3 and Caspase-1 genes

**Figure 4.**
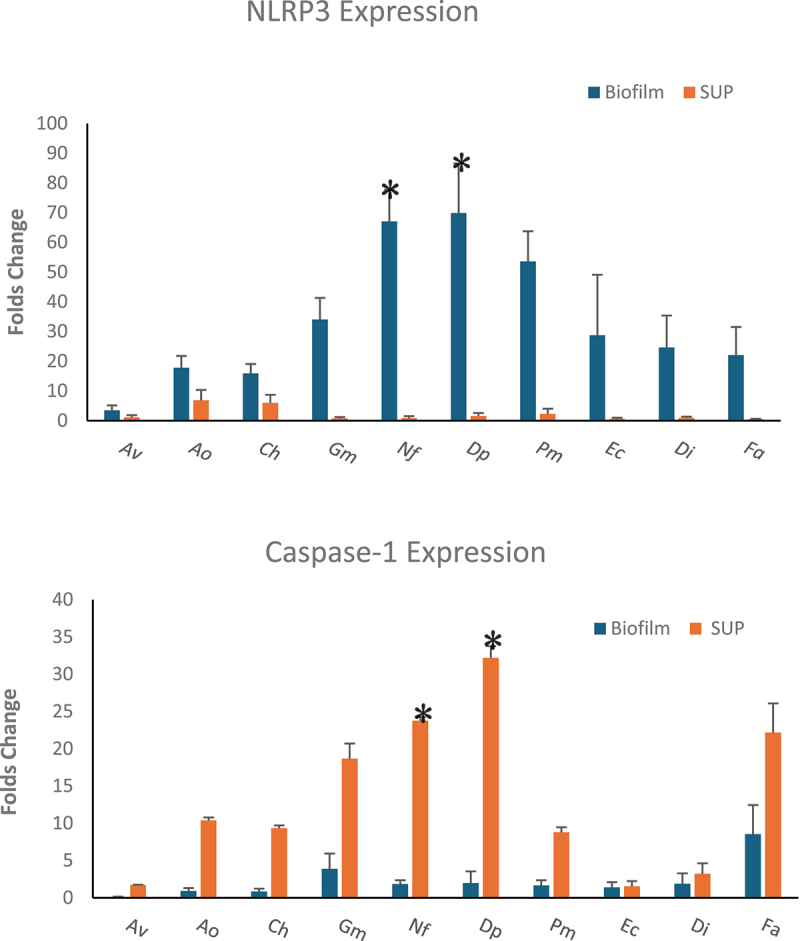
Expression of *nlrp3* and *caspase-1* genes in response to LAB biofilms and supernatants. This figure illustrates the Fold changes in *nlrp3* and caspase-1 gene expression induced by LAB biofilms and supernatants. Biofilms significantly upregulated nlrp3 expression (*p* < 0.001), with *N. flavescens* and *D. pneumosintes* showing the highest increases, while supernatants induced overall a higher caspase-1 expression. *p < 0.001.

The expression of NLRP3 and caspase-1 genes varied significantly between biofilm and supernatant conditions (Figure 4). Biofilms induced the highest NLRP3 expression (*p* < 0.001), with *D. pneumosintes* (69 ± 16-fold) and *N. flavescens* (67 ± 10-fold) exhibiting the most substantial increases. Other species, including *P. micra* (53 ± 10-fold), *G. morbillorum* (34 ± 7-fold), and *E. corrodens* (28 ± 20-fold) also demonstrated significant (*p* < 0.001) fold changes in expression. In contrast, *C. hominis* and other species exhibited more modest fold changes ranging from 3 ± 1.7 to 17 ± 3.9-fold. In supernatants, *A. odontolyticus* (6.8 ± 3.4-fold) and *C. hominis* (5.9 ± 2.7-fold) exhibited the highest NLRP3 expression, while *E. corrodens* (0.59 ± 0.35-fold) and *F. alocis* (0.47 ± 0.17-fold) induced minimal changes.

Av: *A. viscosus*, Ao: *A. odontolyticus*, Ch: *C. hominis*, Gm: *G. morbillorum*, Nf: *N. flaviscens*, Dp: *D. pneuosintes*, Pm: *P. micra*, Ec: *E. corrodens*, Di: *D. invisus*, Fa: *F. alocis*.

For caspase-1, expression levels were generally higher in supernatants than in biofilms. In the supernatant condition, *D. pneumosintes* exhibited the highest fold change (32 ± 3.4-fold, *p* < 0.05), followed by *N. flavescens* (23.7 ± 2.9), *F. alocis* (22 ± 7), and *G. morbillorum* (18.6 ± 4.7). *E. corrodens* exhibited the lowest fold change (1.5 ± 0.6-fold) in caspase-1 expression. In biofilms, *F. alocis* (8.5 ± 3.9-fold) and *G. morbillorum* (3.8 ± 2-fold) displayed the highest fold changes, whereas *A. viscosus* (0.12 ± 0.04-fold) and *C. hominis* (0.8 ± 0.3-fold) showed minimal changes.

## Discussion

The findings of this study provide novel insights into the immunomodulatory potential of low-abundance bacterial species in the oral cavity. Our results demonstrate that biofilms of LAB species are far more potent inducers of pro-inflammatory cytokines (TNF-α, IL-6, IL-1β, and IL-18) compared to their supernatants. Furthermore, the differential activation of caspase-1and NLRP3 expression by biofilms and supernatants highlights the complexity of host–pathogen interactions in the oral cavity and highlights the importance of bacterial growth states in shaping immune responses.

Overall, cytokine stimulation from biofilm supernatants of any of the LAB species was minimal. The immunostimulatory potential of LAB has been studied to some extent. In a study by Han et al. (2022), low-abundance species of the gut microbiome were shown to elicit a strong immunogenic response [9]. Similarly, previous studies on caries-free and caries-active subjects suggested that low-abundance bacteria, such as *Leptotrichia*, *Selenomonas*, *Prevotella_7*, *Veillonella*, and *Kingella*, could serve as potential biomarkers in cases of severe caries [10,24,25]. Furthermore, it has been shown that LAB species significantly contributed to the expression of several genes involved in the antigen presentation pathway and cytokine production [9].

In general, *C. hominis* exhibited the highest relative secretion of pro-inflammatory cytokines, including IL-10, IL-1β, and IL-18, across biofilm conditions. *N. flavescens* and *D. pneumosintes* also showed a strong pro-inflammatory response in both biofilm and supernatant conditions. This supports the current research linking *C. hominis* and *N. flavescens* to oral disease, as studies on *C. hominis* [26] and *N. flavescens* [27] have demonstrated associations with taxa linked to periodontitis using 16S rRNA and metagenomic sequencing techniques [8]. Interestingly, IL-10, an anti-inflammatory cytokine responsible for regulating immune activity and mitigating excessive inflammation [28], was also elevated for *C. hominis*. This finding suggests that *C. hominis* can modulate immune responses through a dynamic shift between pro-inflammatory and anti-inflammatory signaling. Similar immune responses have been observed for bacteria in the oral cavity and other body systems. *Eggerthella lenta*, a low-abundance gut commensal, has opposing effects on immune responses. It promotes T-helper 17 cell activities by reducing the inhibition of the retinoic acid receptor-related orphan receptor gamma t and inducing interleukin-17A. However, it also suppresses T-helper 17 cell differentiation through its metabolites, 3-oxolithocholic acid and isolithocholic acid [4]. In another study, it was shown that after 4 hours, monocytes initially produced pro-inflammatory cytokines such as TNF-α and IL-1 when exposed to bacteria. After a 24-hour incubation period, as the infection progressed, IL-10 was also detected. The authors postulated that TNF-α plays an important role in the induction of IL-10 [29]. In our study, we measured cytokine levels at 24 hours, which may explain the elevated levels of IL-10. Similarly, in supernatants, the relative pro-inflammatory effect for *N. flavescens* and *C. hominis* followed a similar expression pattern like in biofilm conditions. However, the anti-inflammatory effect was less pronounced for both species compared to the biofilm sample.

In contrast, *A. viscosus* and *A. odontolyticus* induced low levels of TNF-α, IL-6, and IL-1β, and higher or similar levels of IL-10 relative to other microorganisms in both biofilm and supernatant conditions. This is consistent with previous findings that *A. odontolyticus* was not associated with clinical signs of periodontal disease in all examined adults [30] and that *A. viscosus* increased significantly in subgingival plaques following periodontal treatment [31]. Similarly, other bacteria linked to periodontal health, such as *D. invisus*, exhibited relatively higher IL-10 secretion, though secretion levels differed between biofilm and supernatant conditions. On the other hand, negligible cytokine stimulation by *F. alocis* could be possibly due to its poor biofilm growth which may be attributed to limited nutrient availability (e.g. arginine, lysine), the absence of synergistic bacteria, acidic pH, oxidative stress, or short incubation time [32].

In our study, biofilms of LAB species induced significantly higher levels of cytokines compared to biofilm-supernatants, highlighting the critical role of biofilm architecture and extracellular matrix components in augmenting immune responses. The exopolysaccharides components act as pathogen-associated molecular patterns (PAMPs), activating immune cells via pattern recognition receptors like Toll-like receptors (TLR) and NOD-like receptors (NLR), leading to strong pro-inflammatory responses [33]. Host pro-inflammatory response to *in vitro* oral biofilms has been extensively studied. Using well-established *in vitro* subgingival and supragingival biofilm models, induction of important cytokines such as IL-1β, TNF- α, IL-8, IL-2, IL-4, etc., has been investigated in detail [34,35]. These studies have unraveled mechanisms of regulation of inflammasome gene expression providing a deeper insight into host response to oral microbial biofilms.

NLRP3, the most extensively studied member of the NLR family, assembles into the NLRP3 inflammasome alongside ASC (Apoptosis-associated Speck-like protein) and Caspase-1. This inflammasome is activated by various pathogen-associated molecular patterns (PAMPs), leading to the secretion of interleukin (IL)-1β and IL-18 [36–39]. These cytokines are key mediators of inflammation and contribute to tissue damage in periodontal disease [37]. In the present study, biofilms consistently induced higher NLRP3 levels, with *C. hominis*, *N. flavescens*, *G. morbillorum*, and *D. pneumosintes* identified as the most potent activators. These findings align with cytokine data, as NLRP3 activation is known to drive the production of IL-1β and IL-18 [38,40]. The NLRP3 activation observed in response to *N. flavescens*, *C. hominis*, and *D. pneumosintes* biofilms suggests that these species may play a significant role in inflammasome-mediated inflammation in the oral cavity. On the other hand, the role of *G. morbillorum* in periodontal disease remains controversial. While some studies have associated *G. morbillorum* with periodontal health and disease [8,41], its pro-inflammatory mechanism is not well characterized. In particular, the mechanisms underlying its potential to activate NLRP3 remain unexplored in the current literature. Further investigation is warranted to explore whether *G. morbillorum* contributes to inflammasome activation and periodontal inflammation.

Interestingly, supernatants induced higher Caspase-1 levels compared to biofilms, suggesting that soluble bacterial components, such as secreted toxins or metabolic byproducts, may preferentially activate Caspase-1. This dichotomy between NLRP3 and Caspase-1 activation highlights the complexity of inflammasome signaling in response to different bacterial stimuli. For example, soluble factors such as bacterial proteases, ATP, and pore-forming toxins have been shown to activate Caspase-1 independently of NLRP3 [42]. This is consistent with the observation that supernatants of *C. hominis*, *N. flavescens*, *G. morbillorum*, and *D. pneumosintes* induced high levels of Caspase-1, indicating that soluble factors from these species are particularly effective at triggering Caspase-1-mediated immune responses.

The mRNA expression data further corroborate the findings on cytokine production and inflammasome activation. Biofilms induced higher NLRP3 expression, whereas supernatants led to greater Caspase-1 expression. This suggests that biofilm-associated factors primarily activate the NLRP3 inflammasome, whereas soluble bacterial components preferentially trigger Caspase-1 activation. The variability in mRNA expression, particularly for *E. corrodens* and *F. alocis*, suggests species-specific differences in inflammasome activation pathways. For example, *E. corrodens* exhibited minimal Caspase-1 expression in both biofilm and supernatant conditions, indicating that this species may employ alternative mechanisms to evade or modulate host immune responses. This is supported by a study showing that *E. corrodens* induces cytotoxic activity in peripheral blood mononuclear cells, which was reduced by neutralizing anti-CD95L antibodies [43], suggesting a possible shift toward FasL-mediated immune modulation.

The high fold change in Caspase-1 expression in the supernatants of *C. hominis*, *N. flavescens*, *G. morbillorum*, and *D. pneumosintes* aligns with studies demonstrating that bacterial supernatants containing secreted virulence factors – such as proteases, lipopolysaccharides (LPS), and other toxins – can activate Caspase-1 through NLRP3 [44,45]. This is particularly relevant in the context of periodontal disease, where Caspase-1-mediated pyroptosis has been implicated in tissue destruction and bone resorption [46].

The findings of this study have important implications for understanding the role of LAB species in oral health and disease. While *C. hominis* and *N. flavescens* are not dominant members of the oral microbiota, their ability to form biofilms and induce robust immune responses suggests that they may contribute to the pathogenesis of oral infectious diseases. This aligns with the polymicrobial synergy and dysbiosis model of periodontal disease, which proposes that low-abundance species can synergize with more abundant pathogens to drive inflammation and tissue destruction [47]. The virulence potential of *N. flavescens* and *C. hominis* extends beyond the oral cavity. *N. flavescens* has been linked to gastritis, with an urease-producing strain isolated from patients [50], and it also induces immune inflammation and mitochondrial dysfunction, leading to metabolic imbalances in colon carcinoma cells [14]. Likewise, *C. hominis*, a member of the HACEK group, is well-documented in the infective endocarditis [51]. Given these pathogenic capabilities, *N. flavescens* and *C. hominis* may also contribute to oral infections, warranting further mechanistic investigations. Moreover, the differential activation of NLRP3 and Caspase-1 by biofilms and supernatants highlights potential avenues for targeted therapeutic interventions. Strategies aimed at disrupting biofilm formation or targeting biofilm-specific components could help mitigate the immunostimulatory potential of these LAB, thereby reducing their contribution to oral inflammatory diseases. The distinct activation of NLRP3 and Caspase-1 by LAB biofilms and supernatants seen in our study aligns with findings in other oral pathogens. For example, *P. gingivalis*, a key periodontal pathogen, activates the NLRP3 inflammasome in macrophages through secreted factors, even without its proteolytic gingipains [52]. Similarly, *P. gingivalis*-driven IL-1β release in human macrophages requires both Caspase-1 and Caspase-4, showing how canonical and non-canonical inflammasome pathways contribute to periodontal inflammation [53]. While our study highlights LAB, these examples demonstrate that diverse oral bacteria, whether low-abundance or dominant, can exploit inflammasome signaling to alter immune responses. Future studies comparing LAB and established pathogens could reveal whether they disrupt oral homeostasis through similar or unique mechanisms.

One limitation of this study is its reliance on *in vitro* models, which, while valuable for controlled experimentation, do not fully replicate the complex host–microbe interactions in the oral environment. Factors such as immune cell diversity, salivary flow, and microbiome composition may influence responses, necessitating *in vivo* validation. In addition, while this study focused on NLRP3 and Caspase-1 activation, other inflammasomes and immune pathways may also contribute to the observed immune response, warranting further investigation into alternative regulatory mechanisms. The specific biofilm components driving inflammasome activation also remain unidentified. Although biofilms induced stronger immune responses than supernatants, the roles of extracellular polysaccharides, adhesins, and bacterial effectors require further clarification. Furthermore, while our biofilm-supernatant comparisons highlight state-dependent immune activation, future studies could explicitly correlate biofilm maturation stages with secreted factor profiles. Additionally, while our volume selection reflects physiological concentrations, future work could explicitly examine dose–response relationships across biofilm maturation stages. Future studies could integrate non-destructive biomass quantification (e.g. extracellular DNA or confocal microscopy) with transcriptomic/proteomic approaches to correlate immune responses with biofilm maturation states and identify key bacterial immunomodulators.

In conclusion, this study demonstrates that low-abundance oral bacteria, particularly *N. flavescens* and *C. hominis*, are potent inducers of inflammatory and inflammasome responses, with biofilms being more effective than biofilm-supernatants in driving cytokine production and NLRP3 activation. These findings highlight the importance of LAB species in inducing immune responses and suggest that they may play a previously underappreciated role in oral inflammation and disease. The differential activation of NLRP3 and Caspase-1 by LAB biofilms and supernatants further underscores the complexity of host–pathogen interactions in the oral cavity. Future studies should explore the specific bacterial components of LAB species responsible for these immune responses and investigate the potential therapeutic targeting of these pathways to mitigate inflammation associated with oral infections.
